# Real-World Results of Curative Open Colorectal Cancer Surgery in Octogenarians: Long-Term Survival Despite High Frailty Burden

**DOI:** 10.3390/medsci14010101

**Published:** 2026-02-19

**Authors:** Stefan Morarasu, Bogdan Condurache, Cristian Ene Roata, Ana Maria Musina, Wee Liam Ong, Gabriel Mihail Dimofte, Sorinel Lunca

**Affiliations:** 1 Grigore T Popa University of Medicine and Pharmacy, Iasi 700115, Romania; bodocon1998@gmail.com (B.C.); cristian.roata@umfiasi.ro (C.E.R.); ana-maria.musina@umfiasi.ro (A.M.M.); william05021990@gmail.com (W.L.O.); mihail.dimofte@umfiasi.ro (G.M.D.); sorinel.lunca@umfiasi.ro (S.L.); 2 2nd Department of Surgical Oncology, Regional Institute of Oncology, Iasi 700483, Romania

**Keywords:** colorectal surgery, colorectal cancer, octogenarians, geriatrics, frailty, oncology

## Abstract

**Background**: Octogenarians represent a rapidly growing subgroup of patients with colorectal cancer, yet evidence guiding perioperative risk stratification and long-term outcomes after major colorectal surgery remains limited. This study aimed to evaluate perioperative and survival outcomes in octogenarians undergoing curative open colorectal surgery. **Methods:** This single-center observational cohort study included consecutive patients aged ≥80 years who underwent curative open colorectal cancer surgery between 2013 and 2024. Frailty was assessed using the 5-item modified frailty index (mFI-5). Postoperative morbidity, 30-day mortality, and long-term overall survival were analyzed. Outcomes were compared between colon and rectal resections. Exploratory discrimination analyses assessed the ability of age, frailty, and major comorbidities to identify postoperative morbidity. Survival was assessed using Kaplan–Meier analysis. **Results:** A total of 112 patients were included (mean age 83.1 ± 2.8 years; 54.5% male), of whom 90.2% were classified as frail (mFI-5 ≥ 1). Overall postoperative morbidity occurred in 41.9% of patients and 30-day mortality was 4.5%. No significant differences in morbidity or mortality were observed between colon and rectal resections. ROC analyses demonstrated limited discriminatory ability for postoperative morbidity across all evaluated variables, with age showing the highest AUC at 0.590. Estimated OS at 1, 3, and 5 years was 81.8%, 72.7%, and 58.2% in non-frail patients and 86.1%, 64.7%, and 47.0% in frail patients, respectively (log-rank *p* = 0.841). **Conclusions**: Major open colorectal surgery in octogenarians is associated with acceptable perioperative morbidity and mortality and favorable long-term survival despite high frailty burden.

## 1. Introduction

Life expectancy has increased substantially across high-income countries, resulting in an ever-growing number of older adults presenting with colorectal cancer (CRC), which is the second most common cancer in octogenarians after lung cancer [1,2]. Octogenarians represent one of the fastest-expanding demographic groups in surgical oncology [3], yet they remain underrepresented in prospective trials and are often excluded from aggressive or potentially curative surgical strategies [4]. Consequently, decision-making in this population is backed up by scarce evidence and mostly reliant on expert opinion [5].

Major colorectal surgery in patients aged 80 years and above is associated with unique perioperative risks due to diminished physiological reserve, higher multimorbidity, altered postoperative recovery trajectories, and increased susceptibility to complications [6,7]. Traditionally, aging was considered a marker of biological decline and used as a selection criterion for major surgery based on results from individual studies showing higher rates of complications in elderly patients [7]. However, other studies have contradicted this, and now it is recognized that age alone is not enough, as many octogenarians have a well-compensated physiological baseline. To better represent biological decline, frailty has emerged as a more accurate predictor of postoperative risk, hospital resource utilization, and long-term survival. The 5-item modified frailty index (mFI-5) is one of the most applied and validated tools in surgical populations, believed to reflect a patient’s biological age with more confidence than age alone [8,9,10,11].

In CRC specifically, outcomes in very old patients appear to be driven by an interplay between tumor factors, competing risks, and postoperative complications. Large observational studies and single-center cohorts suggest that while octogenarians may experience higher overall complication rates, carefully selected older patients can achieve acceptable short-term mortality and meaningful long-term survival after curative surgery [6,12,13]. Importantly, postoperative complications seem to exert a disproportionate effect on longer-term survival in octogenarians, reinforcing that perioperative optimization and complication prevention are central to maximizing benefit from curative surgery in this age group [14,15,16].

Within the realm of colorectal surgery, apart from patient-level risk factors, the type of colorectal procedure, whether right or left colectomy, low anterior resection (LAR), or abdominoperineal resection (APR), is believed to come with various degrees of morbidity [17]. These procedures differ substantially in operative complexity, cardiopulmonary stress, postoperative complication profiles, and implications for functional recovery. However, few studies have systematically compared outcomes across these operative categories specifically within octogenarian cohorts [5,12,18,19,20]. This limitation is particularly relevant in real-world practice, where decision-making must account for both oncologic intent and the distinct recovery demand of colon versus rectal surgery.

Therefore, the present study aimed to evaluate perioperative outcomes (postoperative morbidity, 30-day mortality, length of stay) and long-term overall survival in a real-world cohort of patients aged ≥80 years undergoing elective curative open colorectal cancer surgery, and to compare outcomes between colon and rectal resections. Additionally, we explored the association between frailty and postoperative and survival outcomes in this population.

## 2. Materials and Methods

### 2.1. Design and Setting

This is a single-center, single-department, observational cohort study on consecutive patients diagnosed with colorectal cancer, aged 80 and above, who underwent open surgery at our institution between 2013 and 2024. All patients underwent standard oncological work-up and management based on multidisciplinary meetings. All patients were treated and followed at our institution. Institutional ethics committee approval was obtained, and informed consent was waived due to the retrospective observational design using routinely collected data, in accordance with hospital regulations.

### 2.2. Inclusion and Exclusion Criteria

**Figure 1:**
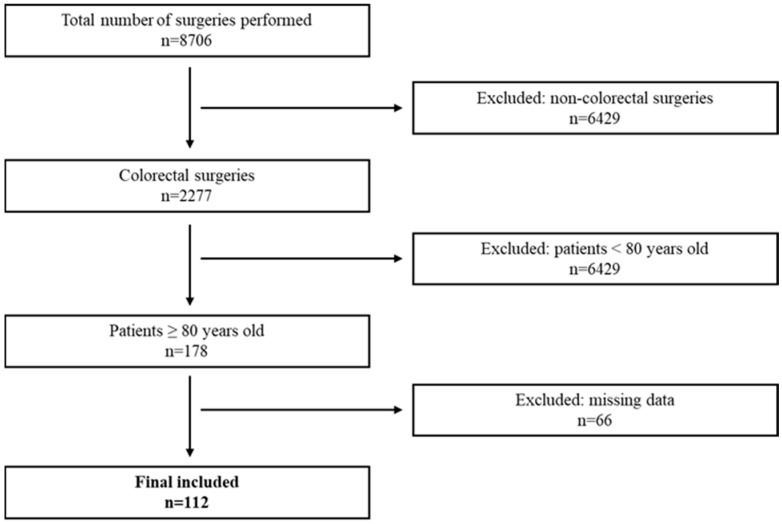
STROBE flow diagram.

The STROBE checklist [21] was adhered to (Figure 1). All octogenarian patients with colorectal cancer who underwent curative surgery were included. Patients who had palliative surgery were excluded. Patients with multiorgan resections were excluded, except for standard en bloc colorectal oncologic resection. Patients with concomitant liver resection for liver metastasis were excluded from this analysis.

### 2.3. Data Analysis

Clinical, pathological, perioperative, and follow-up data were extracted from a prospectively maintained institutional colorectal cancer database and retrospectively analyzed. Data completeness was verified prior to analysis, and only patients with complete datasets for the variables of interest were included in the final cohort. Continuous variables were assessed for normality using visual inspection of histograms. Normally distributed variables are presented as mean ± standard deviation (SD), while non-normally distributed variables are presented as median (interquartile range, IQR). Categorical variables are presented as absolute numbers and percentages. Comparisons between groups were performed according to the type and distribution of variables. For continuous variables, the independent samples *t*-test (pooled variance) was used to compare means between groups and the Mann–Whitney U test for non-normally distributed continuous variables. For categorical variables, the chi-square test was applied when appropriate; when expected cell counts were fewer than five, Fisher’s exact test was used instead. Frailty status was defined using the 5-item modified frailty index (mFI-5). One point was assigned for each of the following preoperative deficits, defined using standardized criteria: functional dependence (defined as requiring partial or total assistance with activities of daily living), diabetes mellitus (DM), chronic obstructive pulmonary disease (COPD), congestive heart failure (CHF), and hypertension (HTA) requiring medication. Patients were stratified into two groups: non-frail (mFI-5 = 0) and frail (mFI-5 ≥ 1). The mFI-5 score was also retained as a discrete variable for exploratory prediction analyses. Other comorbidities, such as chronic kidney disease (CKD) and prior stroke, were not included in the mFI-5 calculation and were analyzed separately as baseline characteristics and candidate predictors. Postoperative outcomes were compared between types of surgery, grouped as colon resections (right colectomy, left colectomy, subtotal/total colectomy) and rectal resections (high anterior resection, low anterior resection, and abdominoperineal resection).

Postoperative morbidity was defined as the occurrence of any medical or surgical complication during the index hospital admission or within 30 days postoperatively. Thirty-day mortality was defined as death from any cause occurring within 30 days of surgery or during the index hospitalization. Receiver operating characteristic (ROC) curve analysis was performed to explore the discriminatory ability of selected preoperative variables for postoperative morbidity. Given that morbidity occurred in 47 patients (42% of the cohort), the outcome did not represent a rare or highly imbalanced event. Therefore, ROC curve analysis was considered an appropriate method for assessing discrimination. For each predictor, the area under the curve (AUC) with corresponding 95% confidence intervals (CI) was calculated. These analyses were conducted in an exploratory manner and were not intended to establish predictive models or clinical cut-offs. Long-term overall survival was defined as the time interval between the date of surgery and death from any cause. Patients alive at the last follow-up were censored on the last known follow-up date. Survival analyses were performed using the Kaplan–Meier method, with survival curves generated according to procedure group (colectomy: right/left/subtotal/total colectomy; rectal resection: high anterior resection, low anterior resection, abdominoperineal resection) and frailty status. Overall survival at 1,3,5 years, standard errors, and 95% confidence intervals were reported. All statistical tests were two-sided, and a *p*-value < 0.05 was considered statistically significant. Statistical analyses were performed using XLSTAT software (v2025.1, Addinsoft, Paris, France) and MedCalc (version 23.4.5).

## 3. Results

### 3.1. Patient Characteristics

A total of 112 octogenarian patients undergoing curative open colorectal surgery were included in the final analysis (Figure 1). The mean age was 83.1 ± 2.8 years, and 61 patients (54.5%) were male. Frailty, defined as a modified frailty index (mFI-5) score ≥ 1, was present in 101 patients (90.2%). The cohort’s frailty distribution was as follows: 11 patients (9.8%) had an mFI-5 score of 0, 19 (17.0%) had a score of 1, 65 (58.0%) had a score of 2, and 17 (15.2%) had a score of 3. Comorbidity burden was substantial. Hypertension requiring medication was the most prevalent condition affecting 99 patients (88.4%), congestive heart failure was present in 81 patients (72.3%), followed by chronic kidney disease in 19 patients (17.0%), diabetes mellitus in 17 patients (15.2%), chronic obstructive pulmonary disease in 11 patients (9.8%), and prior stroke in 3 patients (2.7%). From an oncological perspective, most tumors were locally advanced, with 96 patients (85.7%) presenting with T3–T4 disease and 51 patients (45.5%) having nodal involvement. Distant metastasis at diagnosis was uncommon and identified in one patient (0.9%) (Table 1). Long-course neoadjuvant chemoradiotherapy was performed in 40 patients (35.7%). Characteristics by mFI-5 score (0–3), including age, procedure group, CHF/DM/COPD/CKD, morbidity, and 30-day death, are shown in Table 2.

Regarding surgical procedures, 48 patients (42.7%) underwent right colectomy, 10 patients (8.9%) underwent left colectomy, and 2 patients underwent subtotal and total colectomy (2.8%). Rectal resections accounted for 45.6% of cases, including 27 high anterior resections (24.1%), 19 low anterior resections (17.0%), and 6 abdominoperineal resections (5.4%) (Table 1).

### 3.2. Postoperative Outcomes

Overall postoperative morbidity occurred in 47 patients (41.9%). When stratified by procedure type, morbidity rates were comparable between patients undergoing colon resections (41.7%) and those undergoing rectal resections (42.3%, *p* = 0.849). The overall 30-day mortality rate was 4.5% (n = 5), with no statistically significant difference between colon resections (3.3%) and rectal resections (5.9%, *p* = 0.658). Anastomotic leakage was documented in three patients (2.7%), all occurring after colon resections. Intra-abdominal collections were observed in 3 patients (2.7%), surgical site infections in 19 patients (17%), and Clostridioides difficile infection in 13 patients (11.6%), without significant differences between surgical subgroups. Hospital-acquired infections occurred in 28 patients (25.0%), including hospital-acquired pneumonia in 13 patients (11.6%). Sepsis developed in 8 patients (7.1%), while 9 patients (8.0%) required reintervention (Clavien-Dindo IIIb complications), with no significant variation between colon and rectal surgery groups. Length of stay was similar between colectomy and rectal resection groups (median 12 [IQR 10–14] vs. 13 [10,11,12,13,14,15,16,17,18,19,20,21,22,23,24] days; *p* = 0.401). ICU stay did not differ between groups (median 3 [2,3,4,5,6] vs. 3 [2,3,4,5] days; *p* = 0.608) (Table 3). Another subgroup analysis was performed comparing outcomes between patients who underwent neoadjuvant chemoradiotherapy (CRT) vs. those who did not. Outcomes were similar between the two subgroups (Table 4).

### 3.3. Morbidity Prediction

**Figure 2:**
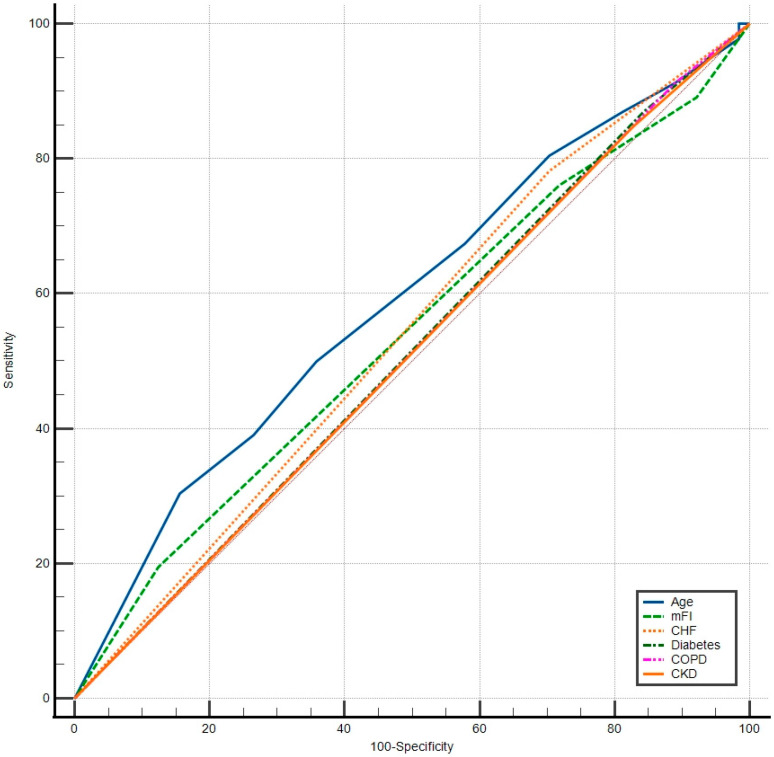
ROC curves comparison in terms of morbidity rate.

Receiver operating characteristic (ROC) curve analyses were performed to evaluate the discriminative ability of age, frailty, and major comorbidities for postoperative morbidity (Figure 2). For postoperative morbidity, none of the evaluated variables demonstrated strong discriminatory performance. The area under the curve (AUC) was highest for age (AUC = 0.590; 95% CI 0.492–0.683), followed by congestive heart failure (AUC = 0.540; 95% CI 0.442–0.635) and by mFI-5 (AUC = 0.538; 95% CI 0.440–0.633). Diabetes, COPD, and chronic kidney disease yielded AUC values close to 0.50, indicating limited predictive accuracy (Table 5). The observed AUC values were modest and associated with relatively wide 95% confidence intervals, reflecting limited precision inherent to the cohort size. None of the evaluated variables demonstrated clinically meaningful discriminatory performance. These findings should therefore be interpreted as exploratory and hypothesis-generating rather than confirmatory. Due to the low number of 30-day mortality events (n = 5), formal discrimination analyses were not performed for this endpoint.

### 3.4. Survival Outcomes

**Figure 3:**
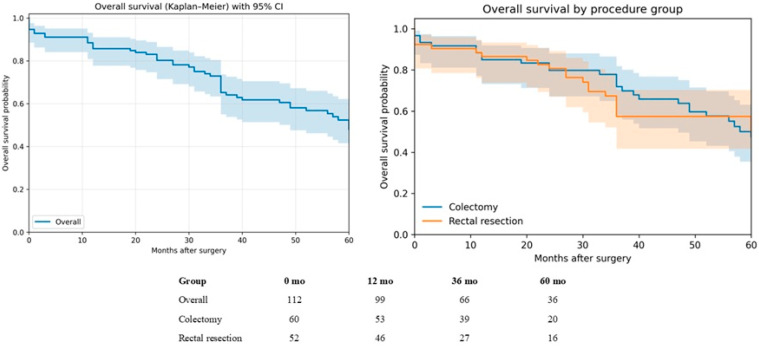
Kaplan–Meier overall survival and by procedure. Note: colectomy: right/left/subtotal/total colectomy; rectal resection: high anterior resection, low anterior resection, abdominoperineal resection. Footnote figure showing the number at risk table.

Kaplan–Meier analysis estimated OS of 85.7% at 1 year, 65.2% at 3 years, and 48% at 5 years. When stratified by procedure category, 5-year OS was 47.5% after colectomy and 50.2% after rectal resection (log-rank *p* = 0.861). (Figure 3 and Table 6).

**Figure 4:**
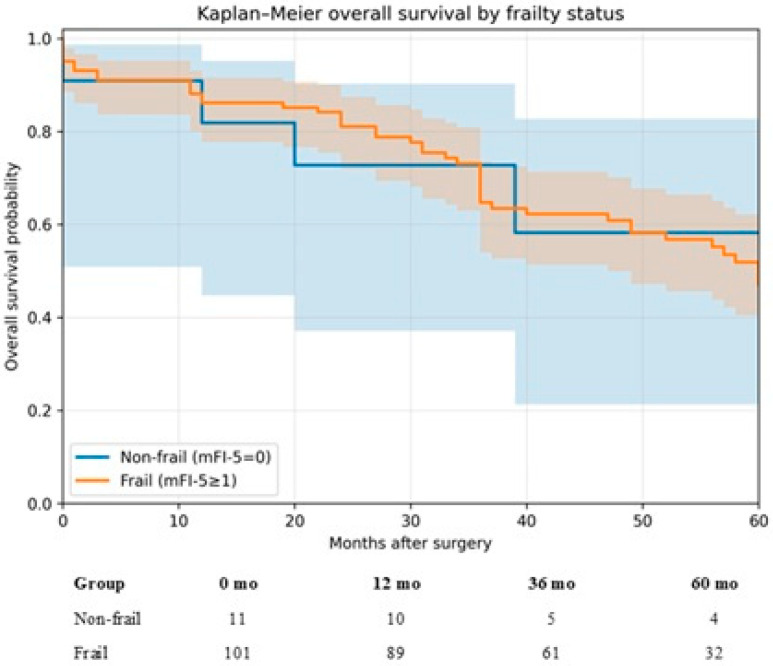
Kaplan–Meier OS survival by frailty status.

**Table 7:** OS survival by frailty status (mFI-5).

Group	N	Deaths	OS 1-Year (12 mo)	OS 3-Year (36 mo)	OS 5-Year (60 mo)
Non-frail (mFI-5 = 0)	11	4	81.8% (44.7–95.1)	72.7% (37.1–90.3)	58.2% (21.3–82.7)
Frail (mFI-5 ≥ 1)	101	45	86.1% (77.7–91.5)	64.7% (54.0–73.5)	47.0% (35.5–57.7)

## 4. Discussion

In this cohort of octogenarian patients undergoing curative colorectal cancer surgery, morbidity, 30-day mortality, and long-term survival were within ranges reported in comparable real-world series. Comparisons between colon and rectal resections did not demonstrate statistically significant differences in postoperative morbidity or mortality; however, these findings should be interpreted cautiously, given the limited sample size and subgroup distribution. Despite analyzing a highly selected subgroup of patients, age, alongside commonly used preoperative risk markers, including frailty and major comorbidities, exhibited limited discriminatory ability to predict postoperative outcomes.

The overall postoperative morbidity rate of 41.9% observed in our cohort is consistent with previously reported rates in elderly colorectal surgery populations, which typically range between 30% and 60%, depending on definitions and case mix [22,23]. In a single-center comparative study, Tondolo et al. [24] reported that elderly patients had a higher overall complication rate than younger patients (47.2% vs. 30.5%), whereas major morbidity (Clavien–Dindo ≥III) was similar (5.7% vs. 6.25%). This aligns with a recurring theme in colorectal surgery: older adults often accumulate more low-grade postoperative events, while the incidence of severe complications may remain comparable when careful patient selection, standardized perioperative care, and enhanced recovery pathways are in place. Across multiple series, the excess burden in older patients is typically driven by medical complications (pneumonia, delirium, cardiac events, and renal dysfunction) rather than procedure-specific surgical failures [13,18,25,26,27,28,29,30]. Consistently, Halabi et al. [31] found that octogenarians experienced more cardiopulmonary complications and required transfusion more often than younger patients. In our cohort, overall complications occurred in 41.9% of patients, yet only 8% developed major complications (Clavien–Dindo IIIb) requiring reintervention. Taken together, these findings suggest that while advanced age is associated with a higher frequency of minor complications and postoperative deconditioning, the risk of serious surgical morbidity (including events such as anastomotic leak) may not be substantially higher once procedure type and comorbidity burden are accounted for. Supporting this interpretation, Høydahl et al. [15] reported a 9.6% major complication rate (Clavien–Dindo ≥III) in a mixed-age cohort and found that, after adjustment for relevant confounders, octogenarians were not disproportionately affected.

The 30-day mortality rate of 4.5% found in our study is in accordance with previous literature [6,22,23]. A tertiary center study from 2019 to 2024 reported a 30-day mortality of only 3.3% among 211 colorectal cancer patients aged ≥75 [16]. Similarly, in an analysis limited to elective surgeries in patients ≥80, the 30-day mortality was 2.5%, statistically no different from that of younger control groups [6]. Some smaller single-institution audits have even reported zero deaths within 30 days in their octogenarian cohorts [13], though this likely reflects the stringent selection of only the very fittest elderly for surgery. Overall, these data emphasize that chronological age alone should not be viewed as a contraindication to major colorectal surgery when careful selection and high-quality perioperative care are applied. What matters most is fitness and the presence or absence of serious complications. In this regard, Weerink et al. [14] showed that octogenarians who develop severe complications have substantially higher mortality in the months following surgery than those with an uncomplicated course. In that cohort, median survival was approximately 13 months in patients with major complications versus 66 months in those without, and postoperative complications were associated with more than a three-fold higher hazard of death after adjustment. This supports the practical conclusion that complication prevention is central to improving survival in vulnerable elderly patients.

Importantly, several studies indicate that if older patients survive the immediate postoperative period, their longer-term cancer-related outcomes may approach those of younger patients with comparable disease characteristics. Høydahl et al. illustrated this by showing that among octogenarians surviving 90 days postoperatively, 5-year relative survival approached that of an age-matched general population (98.7%) [15]. Similarly, registry-based analyses referenced by Grainger et al. suggest that, after recovery from surgery, long-term survival can be comparable to younger cohorts when stratified by stage and comorbidity [16]. These observations reinforce that perioperative risk is “front-loaded,” and that patients who pass through the early hazard window may derive substantial long-term benefit from curative surgery.

Several studies reinforce the concept that, in older adults with colorectal cancer, oncologic control and overall survival may diverge because of competing non-cancer risks. Ogata et al. reported no clinically meaningful age-related differences in disease-free survival for stage I–III disease, despite inferior overall survival in patients aged ≥80, an effect largely explained by non-cancer mortality, which accounted for roughly 70% of deaths in the ≥80 group [6]. Similar observations were described by Passuello et al. [32] and Oh et al. [33]; although octogenarians had lower overall survival, disease-free/recurrence-free survival was broadly comparable after accounting for stage and other confounders. In Ogata’s multivariable analysis [6], frailty, poor nutritional status, and open surgery were associated with non-cancer mortality, whereas cancer-specific mortality was driven predominantly by tumor-related factors. Collectively, these findings suggest that improving outcomes in elderly CRC patients depends not only on oncologic treatment but also on mitigating competing risks through optimization of baseline health.

Accordingly, physiological age, captured by frailty and functional status, often predicts postoperative risk better than chronological age. Frailty is frequent among older CRC patients and consistently identifies individuals at increased risk of complications, functional decline, and death. In cohorts evaluated with comprehensive geriatric assessment, frailty prevalence is substantial (for example, 43% in two octogenarian series summarized by González et al. [7]) and is consistently linked with worse postoperative trajectories (7). Kristjansson et al. [34] reported markedly higher complication rates in frail versus non-frail patients (any complication 76% vs. 48%; severe complications 62% vs. 35%), while Ommundsen et al. [35] found profoundly reduced long-term survival among frail patients (5-year survival 24% vs. 66%, *p* < 0.001). Taken together, these data emphasize that frailty-based risk stratification can be more discriminative than age alone—such that a robust 85-year-old may tolerate surgery well, whereas a frail 75-year-old may not. Incorporating structured frailty assessment (mobility, strength, cognition, nutrition, and functional independence) can therefore refine preoperative decision-making and help target individualized prehabilitation and optimization strategies [36,37]. Interestingly, in our cohort (90.2% frail patients), frailty did not demonstrate strong discriminatory performance for postoperative morbidity (Figure 2). This is not necessarily contradictory to the literature; rather, it highlights a common challenge in real-world surgical risk stratification: single variables (even clinically meaningful ones) rarely predict heterogeneous postoperative endpoints with high accuracy, particularly in selected cohorts and when event counts are low.

Rectal surgery is known to be more technically demanding and often perceived as being associated with a higher risk of complications compared to colon surgery. In the present study, however, morbidity, mortality, length of stay, and ICU stay were comparable between colon resections and rectal resections, including low anterior and abdominoperineal resections. Moreover, in this cohort, 5-year survival was similar for colectomies and rectal resection procedures. This suggests that, in octogenarians deemed fit for surgery, the anatomical extent and technical complexity of the colorectal procedure may be less influential on short-term outcomes than one might have considered. This finding is clinically relevant because it argues against reflexively downscaling curative intent solely because the index operation is rectal rather than colonic, while still emphasizing that careful case selection and standardized perioperative pathways remain central. In a National Cancer Database analysis (2005–2019) including 662,102 colon and 114,460 rectal cancer resections, patients aged ≥80 years had higher short-term mortality after colon surgery (30-day 7.6% vs. 5.9%; 90-day 12.9% vs. 10.3%, colon vs. rectal) (19). Likewise, in a Dutch cohort of operated stage I–III disease (age ≥75 years; 2749 colon and 1053 rectal cancers), postoperative mortality remained higher for colon cancer (30-day 7.5% vs. 3.7%; 1-year 23.2% vs. 20.1%), with greater 1-year excess mortality (16.0% vs. 13.1%) [22].

Beyond the immediate postoperative period, however, differences in outcomes between colon and rectal cancer in the elderly appear to attenuate, and long-term prognosis is increasingly influenced by first-year vulnerability and competing non-cancer mortality. In the Netherlands Cancer Registry (2008–2013), 5-year overall survival was broadly similar by age group (75–84 years: 56% colon vs. 56% rectal; ≥85 years: 35% vs. 38%), yet 1-year excess mortality remained consistently higher for colon cancer (75–84 years: 10.5%; ≥85 years: 17.3%) than for rectal cancer (8.9% and 12.9%, respectively) [20].

Several limitations of this study should be acknowledged. First, the single-center design inherently limits the generalizability of the findings. Second, the retrospective analysis of prospectively collected data introduces an unavoidable risk of selection bias. Third, the study exclusively evaluated open colorectal procedures, as minimally invasive approaches were excluded to preserve cohort homogeneity, considering open surgery is known to have higher risks compared to laparoscopic/robotic surgery, especially in elderly patients. While this enhances internal validity, it limits the applicability of the results to modern practice. The present study should be interpreted primarily as a descriptive real-world cohort analysis rather than a predictive modeling study. The modest sample size and limited number of outcome events restrict the precision of subgroup and discrimination analyses. Consequently, the absence of statistically significant differences between colon and rectal resections, as well as between frailty status, should not be interpreted as evidence of equivalence but rather as reflecting limited statistical power. These findings should therefore be viewed as hypothesis-generating and require confirmation in larger multicenter datasets. The use of ROC curve analysis in this study warrants clarification. Discrimination analysis was restricted to postoperative morbidity, where the event rate was approximately 41%, thereby avoiding the extreme class imbalance that may limit the interpretability of ROC curves. While precision–recall curves are sometimes recommended for rare-event settings, the moderate prevalence of morbidity in our cohort supports the continued appropriateness of ROC-based discrimination assessment. Nevertheless, given the overall sample size and number of events, these analyses were conducted in an exploratory framework. The resulting AUC estimates were modest and accompanied by wide confidence intervals and, therefore, should not be interpreted as evidence of robust predictive performance. Rather, they provide descriptive insight into the limited discriminatory capacity of individual clinical variables in this octogenarian population. Despite these limitations, the study provides valuable insights into real-world outcomes of major colorectal surgery in octogenarians.

## 5. Conclusions

In this single-center cohort of carefully selected octogenarian patients, major open colorectal cancer surgery was associated with postoperative mortality and long-term survival rates comparable to those reported in similar real-world series. While these findings suggest that chronological age alone should not automatically preclude consideration of curative surgery, treatment decisions must remain individualized and based on comprehensive clinical assessment.

## Figures and Tables

**Table 1:** Patients’ Characteristics.

Variables	N (%)/mean (SD)
**Total**	**112 (100%)**
Age	83.1 (2.8)
Males	61 (54.5%)
mFI-5 ≥ 1	101 (90.2%)
Hypertension	99 (88.4%)
CHF	81 (72.3%)
Diabetes	17 (15.2%)
Stroke	3 (2.7%)
COPD	11 (9.8%)
CKD	19 (17%)
**Tumor characteristics**	
T3-4	96 (85.7%)
N+	51 (45.5%)
M1	1 (0.9%)
**Type of procedure**	
Right Colectomy	48 (42.8%)
Left Colectomy	10 (8.9%)
Total/Subtotal colectomies	2 (1.8%)
HAR	27 (24.1%)
LAR	19 (17%)
APR	6 (5.4%)
Neoadjuvant CRT	40 (35.7%)
Diverting loop ileostomy	36 (32.1%)

**Key:** SD, standard deviation; mFI-5, modified frailty index; CHF, congestive heart failure; COPD, chronic obstructive pulmonary disease; CKD, chronic kidney disease; HAR, high anterior resection; LAR, low anterior resection; APR, abdominoperineal resection. CRT, chemoradiotherapy.

**Table 2:** Characteristics by mFI-5 score (0–3).

mFI-5	N	Age Mean (SD)	Colectomy n (%)	Rectal n (%)	CHF n (%)	DM n (%)	COPD n (%)	HTA n (%)	Functional Dependence n (%)	Morbidity n (%)	30 d Death n (%)
0	11	83.2 (4.3)	6 (54.5)	5 (45.5)	0 (0.0)	0 (0.0)	0 (0.0)	0 (0.0)	0 (0.0)	6 (54.5)	0 (0.0)
1	19	82.9 (2.2)	12 (63.2)	7 (36.8)	3 (15.8)	2 (10.5)	0 (0.0)	14 (73.6)	0 (0.0)	6 (31.6)	1 (5.3)
2	65	83.0 (2.7)	33 (50.8)	32 (49.2)	61 (93.8)	11 (16.9)	7 (10.8)	65 (100.0)	1 (1.5)	26 (40.0)	2 (3.1)
3	17	83.6 (2.5)	9 (52.9)	8 (47.1)	17 (100.0)	4 (23.5)	4 (23.5)	17 (100.0)	9 (52.9)	9 (52.9)	2 (11.8)

**Key:** SD, standard deviation; mFI-5, modified frailty index; CHF, congestive heart failure; DM, diabetes mellitus; COPD, chronic obstructive pulmonary disease; CKD, chronic kidney disease; HAR, high anterior resection; LAR, low anterior resection; APR, abdominoperineal resection. Values are n (%) unless stated otherwise. LOS and ICU stay are median (IQR).

**Table 3:** Postoperative outcomes based on the type of procedure.

Outcomes	Total N (%)	Colon Resection N (%)	HAR/LAR/APR N (%)	*p* Value
**Total**	**112 (100%)**	**60 (53.6%)**	**52 (46.4%)**	
Morbidity	47 (41.9%)	25 (41.7%)	22 (42.3%)	0.849
30-day Mortality	5 (4.5%)	2 (3.3%)	3 (5.7%)	0.658
Anastomotic leak	3 (2.7%)	3 (5.0%)	0 (0.0%)	
IAC	3 (2.7%)	2 (3.3%)	1 (1.9%)	
Evisceration	3 (2.7%)	1 (1.7%)	2 (3.8%)	
SSI	19 (17.0%)	10 (16.7%)	9 (17.3%)	
CDI	13 (11.6%)	8 (13.3%)	5 (9.6%)	
HAI	28 (25.0%)	15 (25.0%)	13 (25.0%)	
HAP	13 (11.6%)	7 (11.7%)	6 (11.5%)	
Sepsis	8 (7.1%)	6 (10.0%)	2 (3.8%)	
Reintervention	9 (8.0%)	4 (6.7%)	5 (9.6%)	0.731
LOS	12.5 (10.0–16.0)	12.0 (10.0–14.0)	13.0 (10.0–24.0)	0.401
ICU stay	3.0 (2.0–5.0)	3.0 (2.0–6.0)	3.0 (2.0–5.0)	0.608

**Key:** HAR, high anterior resection; LAR, low anterior resection; APR, abdominoperineal resection; IAC, intra-abdominal collection; SSI, surgical site infection; CDI, clostridium difficile infection; HAI, hospital-acquired infection; HAP, hospital-acquired pneumonia; LOS, length of stay; ICU, intensive care unit; NA, not applicable. Note: Individual complication categories are not mutually exclusive (a patient may have more than one complication). Given the small cell counts for several complications, statistical testing for individual complications is underpowered, and *p*-values are not shown.

**Table 4:** Postoperative outcomes based on CRT status.

Group	Total N (%)	Morbidity N (%)	30-Day Mortality N (%)	Frailty (mFI-5 ≥ 1)	LOS	ICU Stay
**CRT**	40 (36.0%)	15 (37.5%)	3 (7.5%)	37 (92.5%)	20.5 (19.8)	4.4 (4.1)
**No CRT**	72 (64.0%)	31 (43.1%)	2 (2.7%)	63 (87.5%)	15.2 (9.5)	4.9 (4.4)
***p* value**	NA	0.666	0.349	0.759	0.316	0.578

**Key:** CRT, chemoradiotherapy; LOS, length of stay; ICU, intensive care unit.

**Table 5:** ROC curve analysis of each variable and their influence on morbidity.

Variable	AUC	SE	95% CI
Age	0.590	0.0551	0.492 to 0.683
mFI-5	0.538	0.0508	0.440 to 0.633
CHF	0.540	0.0421	0.442 to 0.635
Diabetes	0.513	0.0340	0.416 to 0.609
COPD	0.511	0.0288	0.414 to 0.608
CKD	0.510	0.0358	0.413 to 0.606

**Key:** AUC, area under the curve; SE, standard error; CI, confidence interval; mFI-5, modified frailty index; CHF, congestive heart failure; COPD, chronic obstructive pulmonary disease; CKD, chronic kidney disease.

**Table 6:** Overall survival and OS by procedure at 1, 3, 5 years.

Group	N	Deaths	OS 1-Year (12 mo)	OS 3-Year (36 mo)	OS 5-Year (60 mo)
Overall	112	49	85.7% (77.7–91.0)	65.2% (55.0–73.6)	48.0% (37.1–58.1)
Colectomy	60	27	85.0% (73.2–91.9)	71.9% (58.1–81.8)	47.5% (32.9–60.8)
Rectal resection	52	22	86.5% (73.8–93.3)	57.3% (41.7–70.2)	50.2% (33.8–64.5)

Kaplan–Meier analysis demonstrated similar overall survival between non-frail (mFI-5 = 0; n = 11; death = 4) and frail patients (mFI-5 ≥ 1; n = 101; deaths = 45). Estimated OS at 1, 3, and 5 years was 81.8%, 72.7%, and 58.2% in non-frail patients and 86.1%, 64.7%, and 47.0% in frail patients, respectively. Survival curves did not differ significantly by frailty status (log-rank *p* = 0.841), noting the small size of the non-frail group and corresponding wide confidence intervals (Figure 4, Table 7).

## Data Availability

The raw data supporting the conclusions of this article will be made available by the authors on request.

## Abbreviations

The following abbreviations are used in this manuscript:

APR	Abdominoperineal resection
AUC	Area under the curve
CDI	*Clostridioides difficile* infection
CHF	Congestive heart failure
CI	Confidence interval
CKD	Chronic kidney disease
COPD	Chronic obstructive pulmonary disease
CRC	Colorectal cancer
CRT	Chemoradiotherapy
DM	Diabetus mellitus
HAR	High anterior resection
HAI	Hospital-acquired infection
HAP	Hospital-acquired pneumonia
HTA	Hypertension
ICU	Intensive care unit
IAC	Intra-abdominal collection
IQR	Interquartile range
LAR	Low anterior resection
LOS	Length of stay
mFI-5	5-item modified frailty index
NA	Not applicable
OS	Overall survival
ROC	Receiver operating characteristic
SD	Standard deviation
SE	Standard error
SSI	Surgical site infection
STROBE	Strengthening the Reporting of Observational Studies in Epidemiology
